# Discrimination and Integration of Phonological Features in Children with Autism Spectrum Disorder: An Exploratory Multi-Feature Oddball Protocol

**DOI:** 10.3390/brainsci15090905

**Published:** 2025-08-23

**Authors:** Mingyue Zuo, Yang Zhang, Rui Wang, Dan Huang, Luodi Yu, Suiping Wang

**Affiliations:** 1 Philosophy and Social Science Laboratory of Reading and Development in Children and Adolescents, South China Normal University, Guangzhou 510631, China; mingyuezuo@163.com (M.Z.); 2016022527@m.scnu.edu.cn (R.W.); fandaomaoyan@163.com (D.H.); yuluodi@gzhu.edu.cn (L.Y.); 2 Department of Speech-Language-Hearing Sciences and Masonic Institute for the Developing Brain, University of Minnesota, Minneapolis, MN 55455, USA

**Keywords:** Autism Spectrum Disorder, speech perception, auditory integration, ERP

## Abstract

**Background/Objectives:** Children with Autism Spectrum Disorder (ASD) often display heightened sensitivity to simple auditory stimuli, but have difficulty discriminating and integrating multiple phonological features (segmental: consonants and vowels; suprasegmental: lexical tones) at the syllable level, which negatively impacts their communication. This study aims to investigate the neural basis of segmental, suprasegmental and combinatorial speech processing challenges in Mandarin-speaking children with ASD compared with typically developing (TD) peers. **Methods:** Thirty children with ASD and thirty TD peers will complete a multi-feature oddball paradigm to elicit auditory ERP during passive listening. Stimuli include syllables with single (e.g., vowel only), dual (e.g., vowel + tone), and triple (consonant + vowel + tone) phonological deviations. Neural responses will be analyzed using temporal principal component analysis (t-PCA) to isolate overlapping ERP components (early/late MMN), and representational similarity analysis (RSA) to assess group differences in neural representational structure across feature conditions. **Expected Outcomes:** We adopt a dual-framework approach to hypothesis generation. First, from a theory-driven perspective, we integrate three complementary models, Enhanced Perceptual Functioning (EPF), Weak Central Coherence (WCC), and the Neural Complexity Hypothesis (NCH), to account for auditory processing in ASD. Specifically, we hypothesize that ASD children will show enhanced or intact neural discriminatory responses to isolated segmental deviations (e.g., vowel), but attenuated or delayed responses to suprasegmental (e.g., tone) and multi-feature deviants, with the most severe disruptions occurring in complex, multi-feature conditions. Second, from an empirically grounded, data-driven perspective, we derive our central hypothesis directly from the mismatch negativity (MMN) literature, which suggests reduced MMN amplitudes (with the exception of vowel deviants) and prolonged latencies accompanied by a diminished left-hemisphere advantage across all speech feature types in ASD, with the most pronounced effects in complex, multi-feature conditions. **Significance:** By testing alternative hypotheses and predictions, this exploratory study will clarify the extent to which speech processing differences in ASD reflect cognitive biases (local vs. global, per EPF/WCC/NCH) versus speech-specific neurophysiological disruptions. Findings will advance our understanding of the sensory and integrative mechanisms underlying communication difficulties in ASD, particularly in tonal language contexts, and may inform the development of linguistically tailored interventions.

## 1. Introduction

Autism Spectrum Disorder (ASD) is a neurodevelopmental condition characterized by differences in social interaction, speech communication, and other behaviors, including sensory processing difficulties [1]. Language difficulties are common in ASD but vary widely, influenced by factors such as cognitive abilities, symptom severity, and co-occurring conditions [2]. This heterogeneity underscores the importance of investigating both basic auditory encoding and higher-order integrative mechanisms to better understand the diverse language profiles observed in ASD.

Prior research has linked language challenges in ASD to atypical discrimination of both nonspeech and speech sounds [3,4,5]. Despite growing interest in auditory processing in ASD, most studies have focused on a narrow range of auditory contrasts, often isolating segmental or suprasegmental features without investigating how multiple features are processed together [6]. This limits our understanding of how children with ASD perceive complex, ecologically valid speech signals. To address this, we examine Mandarin Chinese, a tonal language in which lexical meaning is determined by contrasts in consonants (C), vowels (V), and tones (T). This provides a rich, structured context to explore how children with ASD discriminate and integrate multiple phonological features, both in isolation (C, V, T) and in combination (e.g., C + V, V + T, C + T, C + V + T), as they naturally occur in Mandarin words.

However, no single existing theoretical framework directly predicts the exact pattern of differences between children with ASD and typically developing (TD) peers across the seven types of speech contrasts examined in this exploratory study. To address this, we review experimental findings and protocols on the processing of lexical tones, vowels, and consonants as well as complementary theoretical perspectives from existing literature to inform our experimental design. We will also develop theory-driven and data-driven hypotheses and predictions regarding how ASD may influence the neural processing of single- and multi- feature speech contrasts, at both local (segmental) and global (beyond segmental) levels.

### 1.1. Atypical Acoustic Speech Feature Processing in ASD

Speech perception involves the multi-dimensional encoding of acoustic features such as fundamental frequency (F0), voice onset time, formant transition, and formant structure [7,8,9]. To assess how the brain automatically discriminates these acoustic variations, researchers commonly use the mismatch negativity (MMN), an event-related potential (ERP) component that reflects pre-attentive auditory discrimination [10]. MMN is particularly valuable for studying the neural mechanisms underlying early auditory perception. Its key advantage lies in being a “pre-attentive” ERP component, meaning it can be elicited while participants are passively listening, not actively attending to the stimuli. This makes it particularly valuable for research with populations who may have difficulty following complex instructions or sustaining attention, such as infants, young children, and individuals with neurodevelopmental conditions like ASD. Importantly, MMN responses for speech sounds in early childhood have been found to be predictive of subsequent language and reading skills, indicating their relevance for early screening and intervention planning in children at risk for language impairments [11,12].

#### 1.1.1. Lexical Tone Processing

In tonal languages like Mandarin, pitch contours, which are conveyed through F0 variations at the syllable level, create lexical contrasts (e.g., “妈” [mā] with a flat tone means “mother,” while “骂” [mà] with a falling tone means “scold”). In contrast, non-tonal languages (e.g., English) use pitch for prosodic functions like intonation and emphasis [13,14,15]. This distinction is critical for understanding auditory processing in ASD, where research reveals a dissociation between non-speech and speech-related pitch processing. While individuals with ASD often exhibit intact or enhanced sensitivity to simple, non-speech pitch changes (e.g., demonstrating faster reaction times and stronger MMN responses to frequency deviants [16]), they struggle to integrate pitch information into more complex contexts [17,18]. For instance, despite heightened pitch discrimination, individuals with ASD struggle with auditory stream segregation [19], suggesting a bias toward local pitch features at the expense of integrating these features into different voice streams.

This pattern is particularly evident in tonal language studies. Children with ASD show reduced MMN amplitudes in response to Mandarin and Cantonese tone contrasts, even though they exhibit enhanced MMN responses to non-speech pitch deviations [20,21]. They also do not show the typical enhancement of MMN for between-category tone contrasts compared to within-category acoustic differences, indicating difficulties with categorical perception of lexical tones [22]. These findings suggest that while basic pitch detection may be preserved or heightened in ASD, the integration of dynamic F0 contours with phonological and linguistic context is problematic [20,23,24,25]. Thus, the core challenge lies not in detecting pitch changes, but in assigning them linguistic meaning within a speech signal.

#### 1.1.2. Consonant Processing

Unlike suprasegmental features such as lexical tone, consonants are primarily identified by transient acoustic cues such as voice onset time (VOT), formant transitions, burst amplitude, and spectral properties [26]. These rapid, dynamic changes require precise auditory temporal resolution for accurate perception. Kuhl et al. (2005) found that typically developing (TD) children elicited a clear MMN response to the /ba/ vs. /wa/ contrast, whereas children with ASD did not, indicating reduced sensitivity to consonantal contrasts in ASD [27]. This finding has been interpreted as a deficit in early phonetic discrimination. Additionally, children with ASD demonstrate less categorical perception of VOT contrasts for stop consonants, indicating atypical encoding of phonemic boundaries [28]. These perceptual difficulties likely stem from challenges in rapid acoustic discrimination, which relies on fine-grained auditory timing [29,30]. Children with ASD show longer latencies in auditory evoked responses, reduced neural synchrony and lower MMNs [31,32,33]. Such disruptions in neural timing may affect the brain’s ability to parse brief, rapidly occurring cues such as formant transitions or burst timing that are critical for consonant identification.

However, an important caveat arises from a follow-up analysis in which the ASD group was subdivided based on auditory preference [27]. Children with ASD who preferred non-speech stimuli still failed to elicit an MMN, consistent with the initial finding. In contrast, those who preferred motherese (infant-directed speech) showed MMN amplitudes comparable to TD controls. This suggests that individual differences in sensory preference and attentional engagement may modulate neural speech discrimination in ASD.

#### 1.1.3. Vowel Processing

Vowels are primarily distinguished by their first and second formants (F1 and F2). Research shows that children with ASD demonstrate significant differences in vowel processing across multiple levels, ranging from early sensory encoding to higher-order attentional mechanisms [34]. There is evidence that intact MMN responses to vowel contrasts can be found in some individuals with ASD, especially in high-functioning cohorts without language impairment. However, differences in MMN latency, amplitude, or hemispheric lateralization are also reported, particularly in ASD subgroups with more pronounced language difficulties [3,35]. These inconsistencies may stem from variations in the population studied (e.g., language ability), stimulus design, and analysis methods.

Compared to their TD peers, children with ASD show prolonged MMN latencies in response to vowel deviants, indicating delayed neural encoding of relatively steady formant structure [36,37]. Attentional mechanisms are also affected. The P3a component, which follows MMN and reflects automatic attention shift [38,39], is reduced in response to vowel (but not non-speech) deviants in ASD [32,40,41]. This pattern points to a speech-specific problem in attentional engagement, suggesting that while basic acoustic change detection may be preserved, the subsequent allocation of attention to speech-relevant changes is diminished.

### 1.2. Integration Differences in ASD

#### 1.2.1. Cross-Feature Integration in Speech Perception

In natural speech, phonological features do not operate in isolation. Instead, consonants, vowels, and tones interact dynamically to convey meaning [42,43,44]. For example, tone realization in Mandarin depends on concurrent changes in fundamental frequency (F0) and first formant (F1), while stop consonant identification relies on formant transitions into following vowels [45]. This multi-dimensional, temporally overlapping structure of speech requires the auditory system to rapidly integrate acoustic cues across time and frequency domains.

However, individuals with ASD often exhibit disruptions in this integrative process, despite intact or even enhanced sensitivity to isolated acoustic features [46]. A key example comes from Lepistö et al. (2008), who found that while children with ASD showed enhanced neural sensitivity to pitch or phoneme changes presented in isolation, this advantage disappeared when pitch varied within a phoneme, suggesting a failure to extract invariant speech patterns from variable acoustic input [47]. Similarly, ERP and MEG studies show reduced neural differentiation for consonant-vowel transitions [48] and attenuated vowel formant processing in the left auditory cortex linked to poor speech-in-noise performance [49], indicating that the grouping of acoustic elements into coherent percepts is compromised in ASD.

#### 1.2.2. Theoretical Frameworks: EPF, WCC and NCH

Three influential cognitive models, namely, Enhanced Perceptual Functioning (EPF), Weak Central Coherence (WCC), and the Neural Complexity Hypothesis (NCH), offer complementary explanations for these integration difficulties. These frameworks are particularly relevant when considering levels of complexity involving phonological features such as consonants (C) and vowels (V) as local, segmental units, and tone (T), as well as combinations like C + V, V + T, C + T, and C + V + T, as global, integrative features.

WCC posits a cognitive style in ASD that favors local detail processing at the expense of global integration [50]. Applied to speech, this suggests that children with ASD may process individual acoustic cues (e.g., formants) accurately but struggle to bind them into unified linguistic percepts. In contrast, EPF reframes this dissociation: rather than a deficit in global processing, it attributes the pattern to heightened sensitivity to local sensory inputs [51]. This can lead to “perceptual encapsulation,” where low-level features are processed in isolation, interfering with holistic integration. In line with WCC and EPF, individuals with ASD may demonstrate intact or heightened sensitivity to isolated, segmental features such as consonants or vowels while showing weakened neural responses to complex, multi-feature contrasts, including lexical tone or dual/triple feature combinations.

NCH further proposes that individuals with ASD may show intact or enhanced processing of simple stimuli, but progressively impaired responses as stimulus complexity increases [52]. Consistent with the NCH, meta-analytic findings show enhanced pitch discrimination in ASD, but also differences in how pitch is integrated into broader linguistic or musical contexts, suggesting a dissociation between basic auditory encoding and more complex integration processes [18].

The notion of stimulus complexity explains why even segmental features considered “local” like consonants may be more challenging than vowels: consonants require the integration of rapid, transient cues, placing higher demands on temporal parsing and neural synchrony. Indeed, neurophysiological data from non-autistic adults show that stop consonants evoke sparser cortical representations than vowels [53], suggesting they are not “simple” segmental features but rather inherently integrative. Thus, the distinction between “local” and “global” may be less about abstract feature type (e.g., C/V vs. T) and more about integrative cognitive demand. This reframing is critical: it suggests that problematic integration is not a single deficit, but a spectrum of processing burden that escalates with the number and complexity of interacting cues.

#### 1.2.3. The Developmental Imperative: Why Focus on Children?

Studying integration in school-age children (ages 5–10) is essential because this period represents a sensitive window for auditory and language development [54,55]. While foundational auditory plasticity, especially at the level of sensory encoding and language-specific speech representations emerges in infancy and is most pronounced during the first few years of life, higher-order auditory and language-relevant circuits retain plasticity well into childhood, extending through adolescence and adulthood [56,57]. This malleability allows us to capture core processing differences before compensatory strategies or environmental adaptations obscure them.

Moreover, mismatch negativity (MMN) paradigms that do not require focused attention to complete the task are particularly effective in this age group, showing high sensitivity to subtle group differences in auditory processing [20,34]. By focusing on this developmental stage, we can identify early neural markers of integration difficulties—markers that may predict later language outcomes and inform timely, targeted interventions. If integration deficits are rooted in developmental disruptions of neural synchrony or time-binding mechanisms [58,59], then childhood is the optimal window for detecting differences and implementing intervention. Understanding how EPF, WCC, and NCH manifest during this period will not only clarify the cognitive architecture of speech processing in ASD, but also guide the development of culturally and linguistically appropriate strategies to support speech perception and communication.

### 1.3. The Multi-Feature Oddball Paradigm

To investigate how children with ASD process and integrate multiple phonological features across varying levels of complexity, we employ a multi-feature oddball paradigm, an optimized and ecologically grounded variant of the traditional MMN design. Unlike standard paradigms that typically present a single deviant type amidst a high proportion of standard stimuli (80–90%), this approach introduces multiple types of deviants (e.g., tone, vowel, consonant) in an alternating sequence, with the standard stimulus presented only 50% of the time [60]. Each deviant differs from the standard in exactly one dimension, allowing for the efficient and simultaneous assessment of auditory discrimination across multiple speech features within a single, brief session. This design can reduce recording time to approximately 15–20 min while maintaining or even enhancing signal clarity [61].

The multi-feature oddball paradigm has been successfully adapted for speech stimuli by Pakarinen et al. (2009), who used consonant–vowel (CV) syllables as standards, with deviants involving changes in vowel, consonant, syllable pitch (F0), intensity, and duration [62]. This speech-specific version has been validated in both adults and children as young as 6 years old [63], demonstrating reliability and developmental appropriateness. Its ability to probe segmental (consonant/vowel) and suprasegmental (tone) processing within a unified framework makes it ideal for examining the interaction of multiple acoustic cues in a tonal language like Mandarin (See a recent study on Chinese adults [43]). For our purpose, the multi-feature paradigm is uniquely suited to test hierarchical integration. By embedding single-, dual-, and triple-feature deviants within the same experimental context, it allows us to systematically compare neural responses across increasing levels of integrative demand. This enables a direct test of our core hypotheses regarding local vs. global processing and the cumulative burden of feature complexity—questions that cannot be addressed using isolated, single-deviant paradigms.

Finally, the paradigm’s passive listening format minimizes cognitive and behavioral demands, making it highly appropriate for clinical and developmental populations [64,65,66]. It elicits robust ERP responses without requiring active participation, thereby capturing automatic auditory discrimination processes that are foundational to speech perception.

### 1.4. The Current Study

A key innovation of this study is the fine-grained analysis of auditory mismatch responses across varying levels of speech feature complexity. Unlike previous research, which has largely focused on isolated speech elements such as pitch, vowels, or consonants, and thus neglected their dynamic interplay, we aim to investigate how segmental (e.g., consonants, vowels) and suprasegmental (e.g., tone) features are processed and integrated in Mandarin-speaking children with ASD.

We employ a multi-feature oddball paradigm to examine whether children with ASD differ from typically developing (TD) peers in their sensitivity to single-, dual-, and triple-feature speech deviations. Neural responses will be assessed via the mismatch negativity (MMN), focusing on differences in amplitude, latency, and temporal coordination across processing stages. Although the P3a component is sometimes analyzed in the literature, its inconsistent reporting means it will not be a primary focus of our exploratory study.

Our study addresses four key questions:

1. Do children with ASD show diminished MMN responses to speech feature deviations, with greater impairment for complex, multi-feature contrasts?
2. Do ERP differences reflect processing delays that cascade across time, rather than isolated amplitude reductions?
3. How do concurrent changes in tone, consonant, and vowel interact in ERP responses, and do children with ASD show atypical integration patterns in MMN?
4. Given tone’s lexical role in Mandarin, do children with ASD show the most pronounced differences in tone-related conditions, suggesting a language-specific signature of speech processing difficulty?

To address these questions, we adopt a dual-framework approach that distinguishes between theory-driven and data-driven hypotheses. Our theoretical framework integrates three complementary models, WCC, EPF, and NCH, to explain auditory processing in ASD. Together, these models suggest a dissociation as our central hypothesis with specific prediction outlined in Table 1: enhanced or intact MMN responses to single-feature deviants (e.g., consonant or vowel changes), but attenuated or delayed responses to multi-feature deviants (e.g., C + V, V + T, C + V + T), reflecting impaired integration under increasing cognitive load. In this framework, consonants and vowels are tentatively classified as local, segmental features, while tone and multi-feature combinations are treated as global, integrative features.

However, we acknowledge that this local/global conceptual distinction may not fully reflect the neurophysiological reality or complexity of speech perception and thus may not represent the EPF/WCC models accurately. As mentioned earlier, consonant and vowel perception is not purely based on “local” or “isolated” segmental features in the abstract sense as they may involve integration of multiple acoustic cues (e.g., VOT, formant transitions), which can place significant demands on global processing mechanisms. Given the exploratory nature of this study, we treat this framework as provisional—a hypothesis-generating lens to be validated against empirical data.

In contrast to the theory-driven approach, Table 2 specifies predictions based on empirical findings from the MMN literature, focusing on how each stimulus condition may behave in ASD, regardless of theoretical assumptions. When additional features are involved for more complex processing, presumably the MMN differences in amplitude and latency will be magnified in the tired system going from single- to dual- to triple- feature contrasts due to integration difficulties in ASD reported in the literature. While the overall data-driven hypothesis from the MMN literature is that children with ASD will show reduced MMN amplitudes and prolonged latencies across all speech stimulus conditions with effects worsening with complexity, vowel processing stands as a notable exception. As reviewed earlier, some studies have found that children with ASD exhibit prolonged MMN latencies (but not reduced MMN) for vowel stimuli and reduced P3a responses, indicating delayed sensory encoding and impaired attentional orienting to vowel changes. Especially when considering individual variability in language ability or sensory preferences, intact MMN responses may also occur for various speech contrasts (including consonants and vowels) in some subgroups of ASD populations. It is also worth noting that not all MMN studies explicitly report hemispheric data for MMN analyses. Given the growing body of evidence indicates atypical hemispheric specialization for speech in ASD, we tentatively hypothesize that this reduced left-hemispheric MMN advantage (which is not specified in WCC/EPF/NCH models) will be evident across all stimulus conditions, particularly for complex, linguistically relevant contrasts.

## 2. Materials and Methods

### 2.1. Participants

#### 2.1.1. Sample Size Justification and Ethics

The sample size for this study was determined using G*Power (version 3.1) [67]. A priori power analysis was conducted using an MANOVA: Repeated measures, within-between interaction framework as the study compares two groups (ASD vs. TD) with multiple MMN measures. Assuming a relatively large effect size (Cohen’s f = 0.25), the analysis indicated that a total sample size of at least 52 participants (approximately 26 per group) would be required to achieve a power of 0.80 at a significance level of α = 0.05.

This study will be conducted in accordance with the ethical guidelines and approved by the Human Research Ethics Committee for Non-Clinical Faculties at South China Normal University. Written informed consent is obtained from the legal guardians of all participating minors, and assent is secured from the children themselves. Participants are recruited voluntarily through collaboration with the Guangzhou Kangna School and Guangzhou Yongxing School, with explicit inclusion/exclusion criteria to ensure suitability. Risks are considered minimal, as the EEG procedure poses no physical or psychological harm, and participants are free to withdraw at any time without penalty. All EEG and behavioral data collected will be stored securely on institutional servers equipped with firewalls and advanced security protocols compliant with relevant data protection regulations. Hard copies will be securely locked and accessible only to designated research personnel. Data will be anonymized with unique identifiers to protect participant confidentiality. Analysis datasets will be fully de-identified. Secure long-term storage and data sharing practices will adhere strictly to institutional ethics guidelines and the oversight of the Ethics Committee of the School of Psychology, South China Normal University, which reviewed and approved the study protocol.

#### 2.1.2. Inclusion and Exclusion Criteria

ASD Group: A total of 30 children diagnosed with ASD according to DSM-5 criteria (28 males, 2 females; age range: 9–14) will be recruited from Guangzhou Kangna School. Participants met the DSM-5 diagnostic criteria for ASD [1]. The Chinese version of the Gilliam Autism Rating Scale, Second Edition (GARS-2) [68] will be used, which includes three subscales: Stereotyped Behaviors, Communication, and Social Interaction. Scoring is based on DSM-IV-TR [69] and standards from the Autism Society of America (1994). The GARS-2 has shown good validity within the Chinese ASD population [70]. Non-verbal intelligence was assessed using the standard version of the Raven Progressive Matrices, and the scores were within the normal range (percentile ≥ 25).

TD Group: A total of 30 children with no history of neurological or psychiatric disorders (28 males, 2 females; age range: 9–14) will be recruited from Guangzhou Yongxing Primary School. The TD group and ASD group are matched for gender ratio (male: female = 28:2), age (no significant difference between groups as determined by independent samples t-test), and non-verbal intelligence (Raven test scores matched). All participants are native speakers of Mandarin Chinese and right-handed.

Participants will be excluded if they have any of the following conditions: uncorrected hearing impairment, a history of epilepsy or other neurological disorders, hearing abnormalities as indicated by a pure-tone audiometry threshold greater than 25 dB HL, or an inability to cooperate with the experimental tasks, such as excessive head movement or a gaze rate below 70%.

### 2.2. Stimuli, Experimental Procedure and Measurements

#### 2.2.1. Auditory Stimuli and Phonological Manipulations

**Figure 1:**
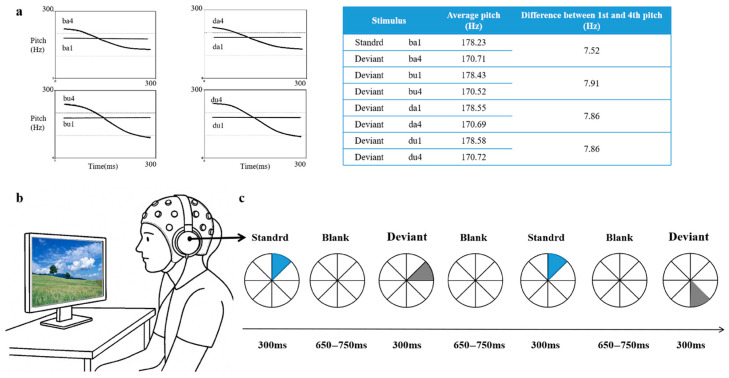
Experimental framework: (**a**) Spectral characteristics of stimuli showing both spectrogram and pitch contour, (**b**) Schematic representation of the experimental apparatus, (**c**) Experiment workflow.

The auditory stimuli consist of eight standardized Mandarin monosyllabic words: /da1/ (level tone), /da4/ (falling tone), /du1/ (level tone), /ba1/ (level tone), /du4/ (falling tone), /ba4/ (falling tone), /bu1/ (level tone), /bu4/ (falling tone). The design systematically manipulates three phonological dimensions: tone (level1 vs. Falling4), vowel (/a/ vs. /u/), and consonants (/d/ vs. /b/). The prepared stimuli were recorded in a soundproof chamber by a native Mandarin-speaking male announcer, using Neundo 4 software (version 4.3.0, Steinberg Media Technologies, Hamburg, Germany) with a sampling rate of 44.1 kHz and a 16-bit resolution in mono. A male speaker was chosen to facilitate consistent F0 control and reduce pitch-related variability across stimuli. Acoustic parameters were standardized using Praat software(version 5.3.51, University of Amsterdam, Amsterdam, The Netherlands) [71]. While duration, intensity, and fundamental frequency are explicitly controlled, other acoustic properties such as voice quality, spectral tilt, and formant trajectories are not formally quantified. The fundamental frequency (F0) setup is as follows: level tone (M = 170 Hz) and falling tone (M = 178 Hz) (see Figure 1a). The duration of each stimulus is fixed at 300 ms, and the sound intensity is normalized to 70 dB SPL. Although we do not formally evaluate additional acoustic parameters such as amplitude envelope, spectral tilt, or formant trajectories, all recordings were produced by the same native Mandarin-speaking male speaker under consistent recording conditions using a clear, child-directed register to minimize within-category variability. The standard stimulus /ba1/ is chosen for its acoustic stability and developmental appropriateness. As a familiar and emotionally neutral syllable with a level tone and simple CV structure, it provides a clear perceptual baseline for detecting phonological deviations.

As this study focuses exclusively on phonological processing of speech sounds, the semantic content of the syllables is not emphasized or manipulated. Each stimulus is a real Mandarin monosyllable with multiple potential lexical meanings, but none are presented with semantic or contextual cues. To minimize potential lexical confounds, all stimuli are selected to be phonotactically legal, emotionally neutral, and matched in syllable structure and duration. Word frequency is not used as an experimental factor, as each syllable in the stimuli is associated with multiple Mandarin homophone candidates. Although all stimuli are phonotactically legal real Mandarin monosyllables, no semantic context is provided, and the task design (passive listening) minimizes lexical access.

The selection of tones, vowels, and consonants is guided by both phonetic salience and linguistic relevance. We use Tone 1 (high-level) as the standard due to its acoustic stability and neutral contour, providing a perceptually robust baseline. Stimuli with Tone 4 (falling) are chosen as deviants for their clear contrast in pitch trajectory, which facilitates reliable MMN detection. The vowel contrast between /a/ and /u/ is selected to capture differences in tongue position and rounding while controlling for stimulus duration and syllable structure. For consonants, /b/ and /d/ differ in place of articulation but share similar voicing, allowing us to examine segmental contrasts while minimizing voicing confounds.

#### 2.2.2. Oddball Paradigm and Experimental Procedure

The experiment adopts a multi-feature oddball paradigm with /ba1/ as the standard stimulus, which appears in 50% of the trials (980 instances). The remaining 50% of the trials (980 instances) consist of seven types of deviant stimuli, each comprising 7.14% of the total. The deviant types include single-feature changes (e.g., from /ba1/ to /ba4/, a change in flat tone to falling tone) and combined-feature changes (e.g., from /da1/ to /bu4/, which has consonant + vowel + tone changes). The full set of stimuli is summarized in Table 3 below:

Stimulus presentation is controlled using the E-Prime 2.0 software. Auditory stimuli are delivered binaurally via a Presonus Firebox audio interface and KOSS PORTA PRO headphones, with the sound intensity calibrated to 65 dB SPL. Each stimulus has a duration of 300 ms, and the stimuli are presented with a randomized stimulus onset asynchrony (SOA) ranging from 950 to 1050 ms, corresponding to an interstimulus interval (ISI) of 650 to 750 ms. The entire experimental session lasts approximately 35 min. To allow participants to adapt to the auditory environment, 20 standard stimuli are presented prior to data collection; these are be excluded from the formal analysis. Participants are instructed to passively listen to the stimuli while watching a self-chosen silent animation throughout the experiment to maintain visual engagement and minimize movement artifacts (see Figure 1a,b).

### 2.3. Data Analysis

#### 2.3.1. EEG Data Acquisition and Preprocessing

Continuous EEG data will be acquired using a 32-channel Brain Products system (10–20 montage) referenced to the left mastoid, with a ground electrode at AFz. Horizontal and vertical electrooculograms (HEOG/VEOG) are additionally used to monitor ocular artifacts. Data are sampled at 1000 Hz with a 0.016–250 Hz bandwidth and impedance maintained below 10 kΩ.

Offline processing in EEGLAB 14.1.0 includes downsampling to 250 Hz, PREP pipeline noise removal [72], 1–30 Hz bandpass filtering, and artifact rejection (±75 µV threshold). Independent component analysis (Fast ICA) will be implemented to remove ocular artifacts, with data re-referenced to the average of all electrodes.

#### 2.3.2. ERP Epoching and Feature Extraction

For each trial, EEG data are segmented into epochs ranging from 100 ms before to 700 ms after stimulus onset. Baseline correction is applied using the pre-stimulus interval (−100 to 0 ms). ERP waveforms are averaged separately for each condition, and the resulting averages are used in subsequent statistical analyses and group comparisons.

#### 2.3.3. Temporal Principal Component Analysis

**Figure 2:**
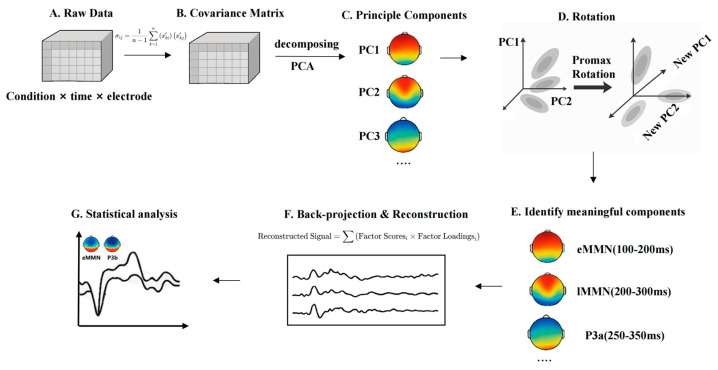
t-PCA pipeline for ERP data in the multi-feature oddball paradigm. (**A**) Data Matrix Setup. The matrix is labeled by Conditions × Time Points × Electrodes, representing trials collected across different stimulus types. (**B**) Covariance Matrix. Covariance matrix is essentially a mathematical tool that helps capture how the data varies over time and across different participants and conditions. (**C**) Principal component analysis. After we obtain the covariance matrix, the next step for t-PCA is to perform eigenvalue decomposition of the covariance matrix to extract the Principal Components from it. These principal components can be seen as new variables extracted from the original EEG signal, which reflect the change pattern of the maximum variance in the data. (**D**) Rotation. The principal components are adjusted using rotation techniques, such as Promax rotation, to improve interpretation. The purpose of the rotation is to make the interpretation between the individual principal components clearer and allow them to have correlations that are more in line with the actual electrical activity of the brain. (**E**) Component identification. By rotating the components, we can begin to identify the individual ERP components that make sense, such as eMMN, lMMN, and P3a. (**F**) Back-Projection and Reconstructed ERP. Components being projected back to ERP space. We multiply the loading matrix of each principal component with the factor scores under each condition to obtain the reconstructed waveform. The reconstructed waveforms contain the specific variation structure of each principal component in the ERP signal, and these waveforms can be interpreted in µV units, like traditional ERP waveforms. (**G**) Amplitude Extraction and Statistical Comparison. This final panel shows mean amplitude extraction from the reconstructed ERP data and reflects statistical testing (e.g., *t*-tests, ANOVA) to assess group differences or condition effects.

Given the use of a multi-feature oddball paradigm in the present study, the ERP components elicited by different deviant types are likely to overlap temporally, making it challenging to isolate the MMN using conventional fixed-window analysis. Prior studies have successfully applied temporal principal component analysis (t-PCA) to decompose overlapping ERP components and better isolate specific responses such as the MMN in oddball paradigms [73,74]. To address this issue, this study will employ t-PCA, a data-driven approach to decompose the ERP signal into latent temporal components. t-PCA enables the extraction of principal components that account for the majority of variance in the time domain, and through rotation and back-projection, allows psychologically meaningful components to be reconstructed in ERP space [75]. Compared to traditional averaging methods, t-PCA offers improved component separation, higher signal-to-noise ratio, and greater sensitivity in detecting condition-specific effects—advantages particularly critical for paradigms involving multi-dimensional speech feature processing (see Figure 2).

To extract multiple ERP components elicited by the multi-feature oddball paradigm, including early mismatch negativity (eMMN), and late mismatch negativity (lMMN), we decompose the time-domain covariance structure of the ERP data and identifies latent temporal components that account for the greatest proportion of variance in the signal.

t-PCA is applied across all conditions and participants, using the covariance matrix and Promax oblique rotation to enhance interpretability and allow for potential correlations among components. Component selection was guided by three criteria: (1) latency and temporal distribution of the component; (2) polarity and waveform characteristics; and (3) topographic distribution across the scalp. Based on previous literature and the typical temporal-spatial profiles of the target components, the following components are identified:

- eMMN: Early mismatch negativity (~100–200 ms; Fz, FCz, Cz)
- lMMN: Late mismatch negativity (~200–300 ms; Fz, FCz, Cz)
- P3a: Fronto-central positivity (~250–350 ms; Fz, FCz, Cz)

Once identified, components are back-projected into ERP space by multiplying temporal loadings with corresponding factor scores for each condition and participant. This is achieved by multiplying the component loadings with the corresponding factor scores for each condition and participant and reprojecting the resulting signal onto the original time axis. The reconstructed waveforms retain the component-specific variance structure while being interpretable in microvolt (µV) units, to facilitate traditional ERP analysis. For each reconstructed component (eMMN, lMMN, and P3a), mean amplitudes are calculated over representative electrode sites within the corresponding time windows. These values are then used in statistical analyses to assess condition effects and group differences in auditory feature processing. Importantly, we selected t-PCA because it enhances sensitivity to subtle neural effects by disentangling overlapping ERP components, isolating latent temporal patterns that may be masked in raw waveforms, and reducing noise through variance-based decomposition [76]. This is particularly critical in multi-feature paradigms, where temporal overlap between components (e.g., eMMN, lMMN, P3a) can obscure condition- and group-specific neural differences.

#### 2.3.4. Representational Similarity Analysis

**Figure 3:**
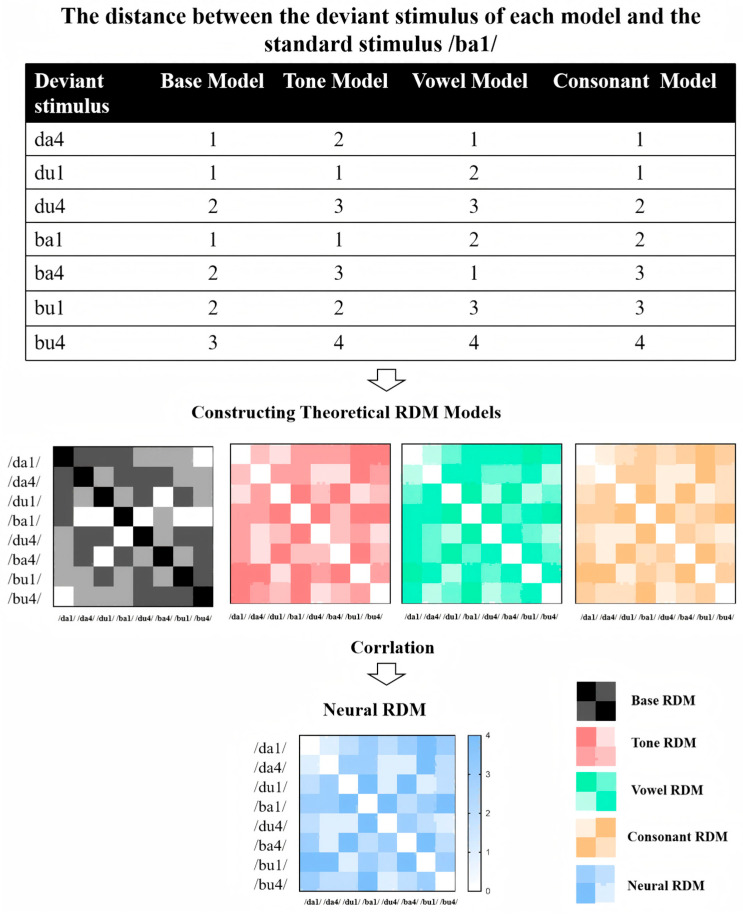
Workflow of RSA applied to ERP data from a multi-feature oddball paradigm.

Representational Similarity Analysis (RSA) has been effectively applied in prior studies to decode different linguistic feature representations, such as decoding multidimensional phonological representations and feature integration in language processing [77,78,79]. To investigate how different speech features (tone, vowel, consonant) are neurally represented and whether individuals with ASD exhibit altered feature integration, we perform RSA based on ERP data. Integration Complexity Models are constructed to quantify the dissimilarity stimuli (see Figure 3). Each model operationalizes dissimilarity between stimulus pairs as a function of changes in three phonological features: Tone, Vowel, and Consonant. The models differ in their weighting schemes to emphasize specific features, reflecting theoretical assumptions about their relative perceptual or cognitive salience. Below is a detailed description of each model:

##### Step 1: Constructing Theoretical RDM Models

- Base Feature-Count Model

This baseline model assumes equal perceptual salience across all three features. Dissimilarity between two stimuli is defined as the sum of the number of altered features. Specifically: Tone, Vowel, or Consonant change contributes a unit weight of 1. For example, a stimulus pair differing in both Tone and Vowel (e.g., /da1/ vs. /du4/) receives a dissimilarity value of 2, while a pair differing in all three features (e.g., /da1/ vs. /bu4/) results in a value of 3. This additive framework generated a symmetric 8 × 8 RDM, with diagonal values (self-comparisons) set to 0 and off-diagonal values ranging from 1 to 3.

- Tone-Weighted Model

To test the hypothesis that Tone carries higher perceptual salience in speech processing, this model assigns a doubled weight (2) to Tone changes, while maintaining unit weights (1) for Vowel and Consonant changes. Dissimilarity values are calculated as follows: A Tone change alone (e.g., /da1/ vs. /da4/) contributes a dissimilarity of 2. A combination of Tone with another feature (e.g., Tone + Vowel: /da1/ vs. /du4/) results in a summed value (2 + 1 = 3). Full feature changes (Tone + Vowel + Consonant) yield a maximum dissimilarity of 4 (2 + 1 + 1). This model’s RDM retains symmetry but amplifies dissimilarities involving Tone changes compared to the base model.

- Vowel-Weighted Model

This model prioritizes Vowel changes with a weight of 2, hypothesizing that vowel identity dominates perceptual discrimination. Tone and Consonant changes retain unit weights (1). Key calculations included: A Vowel-only difference (e.g., /da1/ vs. /di1/) produces a dissimilarity of 2. Combinations involving Vowel changes (e.g., Vowel + Consonant: /da1/ vs. /bu1/) generate higher values (2 + 1 = 3). Triadic feature changes (Tone + Vowel + Consonant) reach a dissimilarity of 4 (1 + 2 + 1). The RDM structure emphasizes vowel-related distinctions while preserving additive feature contributions.

- Consonant-Weighted Model

Mirroring the Vowel-Weighted Model, this framework assigns a weight of 2 to Consonant changes, reflecting a theoretical focus on consonantal salience. Tone and Vowel changes are weighed as 1. Dissimilarity computations included: A Consonant-only difference (e.g., /da1/ vs. /ba1/) is assigned a value of 2. Consonant-involving combinations (e.g., Consonant + Tone: /da1/ vs. /ba4/) yielded summed values (2 + 1 = 3). Full feature changes result in a maximum dissimilarity of 4 (1 + 1 + 2). This model’s RDM highlights dissimilarities driven by consonant alterations.

##### Step 2: Calculating Neural RDM

Neural RDMs were computed based on ERP data, focusing on specific time windows (e.g., eMMN, lMMN, P3a). For each stimulus, the mean amplitude in the relevant time window was extracted. The neural dissimilarity between each pair of stimuli was quantified using either Euclidean distance or Pearson correlation. This process was repeated for each time window of interest, resulting in a series of neural RDMs.

##### Step 3: Comparing Neural RDM with Theoretical Models

To assess how well neural responses align with theoretical models, we compute Spearman’s rank correlation between each neural RDM and the candidate theoretical RDMs. This comparison is performed separately for each time window (eMMN, lMMN, P3a). For group-level comparisons, we conduct the RSA separately for individuals with ASD and TD groups to examine potential differences in how speech features are neurally represented. We use permutation testing combined with cluster-based correction to control multiple comparisons and assess significant differences between groups.

These models assume that phonological features contribute additively to auditory dissimilarity, consistent with the view that early auditory encoding tracks separable acoustic dimensions (e.g., pitch, formant structure, place of articulation) without strong nonlinear interactions. The use of weighted models reflects the hypothesis that tone, vowel, or consonant cues may be differentially salient in perception—especially in tonal languages like Mandarin. While interactive or nonlinear RDMs could in principle be constructed (e.g., hierarchical cue interactions or phonological feature geometry), they require stronger theoretical constraints and larger datasets to validate. Thus, the current additive scheme balances theoretical interpretability, perceptual relevance, and statistical robustness. We employed RSA because it extends beyond univariate amplitude analyses by leveraging multivariate patterns of neural responses, allowing detection of distributed representational differences even when traditional ERP measures yield weak or non-significant effects. This approach is highly suitable for multi-feature paradigms, as it can capture subtle shifts in neural encoding across complex feature combinations and evaluate their alignment with theoretically derived perceptual models [80].

## 3. Predictions Based on Theory- and Data- Driven Perspectives

To guide our analysis and interpretation, we present two complementary sets of predictions derived from distinct sources: (1) a theory-driven framework integrating EPF, WCC, and NCH; and (2) a data-driven framework grounded in the empirical MMN literature. This approach ensures that our expectations are both conceptually motivated and empirically constrained. Predictions are formulated for two levels of analysis: temporal principal component analysis (t-PCA) and representational similarity analysis (RSA). t-PCA will be used to decompose the ERP signal into temporally distinct components (e.g., early MMN, late MMN), from which amplitude and latency estimates are derived. RSA will assess the structure of neural representations across conditions.

### 3.1. Theory-Driven Predictions (Based on EPF, WCC, and NCH Frameworks)

Here, we make some predictions from the tentative local vs. global processing framework (Table 1), which integrates the core tenets of EPF (enhanced local processing), WCC (impaired global integration), and NCH (escalating deficits with complexity). We focus on the eMMN and lMMN components and treat this framework as provisional and testable, acknowledging that the local/global distinction may not fully capture the neurophysiological reality of speech perception.

#### 3.1.1. t-PCA-Level Predictions (P)

P1 (Component Variance): The proportion of variance explained by temporally decomposed MMN components (eMMN, lMMN) will be lower in the ASD group for multi-feature deviants (V + T, C + T, C + V + T), reflecting less structured neural responses under high integrative demand.

P2 (Factor Scores): Factor scores for MMN-related components will be significantly reduced in the ASD group, particularly for tone-involving conditions (T, V + T, C + T, C + V + T), consistent with a global integration deficit.

P3 (Temporal Dynamics): The ASD group will show a delayed peak in late MMN (lMMN) components across all conditions, with the greatest delay observed for triple-feature deviants, supporting NCH’s prediction of cascading processing delays.

#### 3.1.2. RSA-Level Predictions

P4 (Representational Structure): Neural representational dissimilarity matrices (RDMs) in the ASD group will show weaker differentiation between multi-feature deviants (e.g., C + V + T vs. standard) compared to TD children, particularly in the lMMN time window.

P5 (Integration Failure): The fit between neural RDMs and a theoretical “Integrated Features” model (which assumes synergistic processing of C, V, T) will be poorer in the ASD group, especially for V + T and C + V + T conditions.

P6 (Local Bias): The fit between neural RDMs and a “Segmental-Only” model (prioritizing C and V) will be stronger in the ASD group than in TD children, reflecting a local processing bias.

### 3.2. Data-Driven Predictions (Based on MMN Literature)

Predictions are also derived for the t-PCA and RSA analyses from empirical findings across multiple studies (Table 2). They reflect general patterns in the literature and serve as a baseline against which theoretical predictions can be evaluated. They do not assume a local/global dissociation but rather a graded, complexity-dependent deficit.

#### 3.2.1. t-PCA-Level Predictions

P7 (Amplitude Attenuation): Across all conditions (with the exception of vowel deviation), the ASD group will show lower factor scores for MMN components, with the most severe reductions in multi-feature conditions.

P8 (Latency Prolongation): Peak latencies of eMMN and lMMN components will be systematically delayed in the ASD group, with delays increasing as feature complexity rises.

P9 (Component Blurring): The temporal separation between eMMN and lMMN components will be less distinct in the ASD group, suggesting a breakdown in the normal cascade of auditory processing stages.

#### 3.2.2. RSA-Level Predictions

P10 (Weaker Representations): Neural RDMs in the ASD group will show lower overall dissimilarity between deviants and standards across all conditions, with the greatest reduction in C + V + T.

P11 (Atypical Weighting): The fit between neural RDMs and a “Complexity-Weighted” model (which predicts linear degradation with added features) will be stronger in the ASD group than in TD children.

P12 (Hemispheric Asymmetry): Representational dissimilarity in the left hemisphere will be disproportionately reduced in the ASD group, particularly for tone-involving and multi-feature conditions, reflecting a lack of typical left-hemisphere specialization for speech.

The dual-prediction framework allows us to test competing accounts of speech processing in ASD. The predictions are focused on measurable outcomes (overall MMN amplitude, latency, and representational structure) rather than on speculative subcomponents. While we will use t-PCA to decompose the ERP signal and ensure accurate extraction of the MMN, we do not make strong a priori predictions about the distinct roles of early vs. late MMN due to the lack of specific empirical evidence in the ASD literature for multi-feature speech paradigms. Instead, any differences in the temporal dynamics of the MMN will be explored post hoc and reported as exploratory findings, not as confirmatory predictions. By grounding our predictions in established frameworks and focusing on robust, falsifiable outcomes, we ensure a transparent and rigorous evaluation of how Mandarin-speaking children with ASD process and integrate speech features.

## 4. Expected Results

Recruitment began during the third quarter of 2024 and is expected to run for 6 to 9 months, with final data collection anticipated within the first quarter of 2025. Data analysis and interpretation should be completed by the final quarter of 2025.

Upon completion of this study, the expected results will (1) identify neural response patterns derived from temporally decomposed components (eMMN, lMMN) that differentiate ASD and TD groups, particularly highlighting reduced variance explanation and weakened factor scores in the ASD group; (2) clarify differences in single-feature discrimination and multi-feature integration between the two groups, with specific emphasis on diminished sensitivity to increasing feature complexity in ASD, with the most pronounced group differences observed in dual- and triple-feature condition; (3) evaluate the fit of theoretical models of speech integration using Representational Similarity Analysis (RSA); and (4) reveal temporal dynamics of neural integration by examining how the similarity between neural RDMs and theoretical models evolves across the MMN time window. We expect that neural representational structures in the TD group will align more closely with an “Integrated Features” model, while the ASD group may show a stronger fit with a “Segmental-Only” model, supporting a local processing bias. We also expect that group differences in representational structure will become more pronounced in the late MMN (lMMN) period, reflecting delayed or disrupted higher-order integration in ASD. These results will provide a comprehensive, multi-level understanding of how children with ASD process and integrate speech features in a tonal language context.

## 5. Discussion

This research protocol is designed to investigate how Mandarin-speaking children with ASD process and integrate multiple phonological features (consonants, vowels and lexical tones), across varying levels of complexity. As the data are not yet fully analyzed, the purpose of this discussion is not to present conclusions, but to contextualize the study’s design, its potential contributions, and the interpretive frameworks that will be used to evaluate the findings once data collection and analysis are complete.

The central innovation of this research lies in its tiered, multi-feature oddball paradigm, which allows for the systematic comparison of neural responses to single-, dual-, and triple-feature speech deviations within a single, efficient experimental session. This approach moves beyond the traditional focus on isolated acoustic contrasts and instead probes the dynamic integration of segmental and suprasegmental cues—a process fundamental to natural speech perception, especially in tonal languages like Mandarin, where pitch variations directly determine lexical meaning.

The pattern of results will be evaluated against two complementary sets of predictions. If the theory-driven framework (EPF/WCC/NCH) is supported, we expect to see a dissociation: relatively intact or enhanced neural responses (e.g., MMN amplitude, factor scores) to single-feature deviants (e.g., consonant-only, vowel-only), but attenuated or delayed responses to multi-feature deviants (e.g., V + T, C + V + T). This pattern would align with models that posit a cognitive style in ASD characterized by heightened local sensitivity paired with impaired global integration. It would suggest that children with ASD can detect individual phonetic cues but struggle to bind them into unified, linguistically meaningful percepts. If the data-driven framework (MMN literature) is supported, we expect to see a graded, complexity-dependent deficit: reduced MMN amplitudes and prolonged latencies across all conditions, with the most severe effects in multi-feature and tone-involving contrasts. This would be consistent with the Neural Complexity Hypothesis and would suggest a more general processing burden in ASD, where increasing feature complexity leads to cumulative degradation in neural responsiveness, regardless of a local/global distinction. If the results reveal a mixed or unexpected pattern (e.g., no group difference in tone processing), this would highlight the heterogeneity within the ASD population and challenge the universality of current models. Such findings would prompt a re-evaluation of assumptions about what constitutes “local” or “global” processing and could point to subgroup-specific profiles (e.g., based on language ability or sensory preference) that are not captured by broad theoretical frameworks.

A key aspect of our analysis is the inclusion of hemispheric asymmetry as a data-driven prediction. While EPF, WCC, and NCH do not explicitly predict reduced left-hemispheric specialization, the MMN literature suggests that children with ASD may show less lateralized or right-shifted MMN responses to speech sounds. If this is confirmed, it would provide critical evidence for atypical neural organization in ASD, one that may underlie broader difficulties in processing rapid, temporally precise linguistic cues.

We employ two analytical approaches, temporal PCA and RSA. t-PCA decomposes the ERP waveform into latent components (e.g., early and late MMN), isolating overlapping neural responses and increasing sensitivity to group differences, particularly valuable in pediatric data where peak latency and morphology can vary. By extracting factor scores, we can compare the strength and timing of neural responses across conditions and groups. For instance, if two ERP components (such as an early and late MMN) overlap in the 200–300 ms range, t-PCA can help disentangle them into separate factors, allowing us to assess each one’s magnitude in ASD vs. TD children.

In contrast, RSA examines the multivariate structure of neural representations. It quantifies the dissimilarity between brain responses to different stimuli, creating a representational dissimilarity matrix (RDM). We compare empirical RDMs to theoretical models (e.g., “Integrated Features” vs. “Segmental-Only”) to test whether children with ASD represent speech features differently. This approach moves beyond univariate amplitude/latency measures to assess how features are bound into coherent percepts. For example, one model RDM might predict that changing the lexical tone produces large neural dissimilarity (if tone is a salient feature) whereas changing the consonant with the same tone produces a smaller dissimilarity. Another model might predict that any two-feature change (tone + vowel together) yields a bigger neural distance than single-feature changes. We will correlate these model RDMs with the empirical RDMs derived from the EEG data for each group. If TD children’s neural patterns correspond closely to the model emphasizing multi-feature integration, but ASD children’s patterns do not, this would directly support the idea that the ASD group encodes speech features differently. In essence, RSA will tell us how information is represented in the neural activity of each group—whether the brain treats combined feature changes as the sum of their parts or as a unique, salient event, and how this differs between ASD and TD. This multivariate approach is valuable because it moves beyond examining individual ERP peaks and considers the holistic neural response to speech stimuli as a pattern that can be compared to cognitive models.

While the multi-feature oddball paradigm is well-validated in developmental and clinical populations, we acknowledge that detecting fine-grained differences in MMN is challenging. Our analytical approach is designed to maximize sensitivity, but the results must be interpreted within the context of known variability in ASD and the inherent limitations of EEG. Foundational studies [62,81] demonstrated that multi-feature paradigms efficiently detect various speech-sound differences with comparable or superior sensitivity to traditional paradigms, using shorter recording times. More recent work [1,2] has refined these paradigms to capture fine-grained phonetic and prosodic contrasts, sensitive to individual variability in language and musical experience. Critically, studies applying this approach in children at risk for dyslexia [63], children with Asperger syndrome [82], and very young children or infants [83,84], confirm the method’s reliability across ages and populations. Cross-linguistic applications [85] and recent work on emotional prosody [65,86] further demonstrate its feasibility in capturing subtle speech-feature processing differences across linguistic and developmental contexts. Building on this empirical foundation, we employ advanced analytic approaches (t-PCA, RSA) to further enhance sensitivity by isolating latent neural components and distributed representational patterns, thereby increasing the likelihood of detecting group-level differences even when univariate effects are small.

Our carefully matched ASD and TD sample allows us to examine how factors like language ability, symptom severity, and developmental stage modulate neural processing. Testing these predictions in Mandarin, a tonal language where pitch is lexically meaningful, enables a critical test of whether atypical auditory processing in ASD stems from integration deficits (WCC) or enhanced feature detection (EPF)—a distinction with direct implications for intervention. While both EPF and WCC predict reduced responses to multi-feature deviants, they diverge on single-feature processing: EPF predicts enhanced or intact responses, while WCC does not. However, we acknowledge that the existing MMN data suggest that the notions of local vs. global and segmental vs. suprasegmental processing may be oversimplistic. The outcomes may reflect additive or interactive influences among different phonological features.

Regardless of the specific outcomes, this study will provide valuable insights into the neural mechanisms underlying speech perception differences in ASD. If integration deficits are confirmed, it could inform the development of culturally and linguistically tailored interventions that explicitly train children to bind acoustic cues. If local processing advantages are found, interventions could leverage these strengths to support language learning. Furthermore, by focusing on a tonal language, this research addresses a significant gap in the literature, which has largely been based on non-tonal languages. Findings may reveal whether the unique demands of tonal processing amplify or mitigate the typical auditory processing profile in ASD.

In summary, this study protocol combines a sensitive EEG paradigm with advanced analyses to investigate whether atypical speech processing in ASD arises from disrupted feature integration. Our protocol and proposed analyses are intended to contribute much-needed data that can help clarify which aspects of speech sound processing pose the greatest challenges for children with autism. Findings may inform culturally tailored interventions and lay the groundwork for using neural signatures as biomarkers for early language risk.

## 6. Limitations and Future Directions

Several limitations should be acknowledged. First, as with many ERP studies involving clinical populations, sample sizes, particularly within the ASD group, may limit statistical power, especially for detecting nuanced interaction effects. Mitigation strategies include the use of permutation-based statistical corrections and robust within-subject analyses. Second, ERP morphological variability across participants may complicate t-PCA component matching, potentially affecting component stability and RDM construction consistency. Efforts are made to standardize preprocessing and component validation procedures. Third, theoretical RDMs employed in RSA may oversimplify the complex acoustic–phonetic realities of natural speech, potentially affecting ecological validity. Future work could refine these models to encompass finer-grained acoustic and linguistic parameters.

Furthermore, the complex interactions among phonological features (tone, vowel, consonant) may exceed the explanatory scope of current cognitive frameworks such as EPF and WCC. Our study is exploratory in nature, designed not to definitively test these broad theories in their entirety, but to derive and evaluate specific, constrained predictions about local versus global auditory encoding. Findings should therefore be interpreted as preliminary insights into neural processing differences, rather than conclusive evidence for or against any single theoretical account.

Additionally, while the multi-feature oddball paradigm enables precise manipulation of phonological features, it uses isolated syllables in a passive listening context, which lacks the temporal, syntactic, and semantic richness of natural communication. We acknowledge that this design does not fully capture real-world language processing and is not equipped to test high-level cognitive models (e.g., Bayesian Predictive Coding [87,88,89]) in their complete form. Instead, it provides a controlled, developmentally sensitive window into early auditory integration. Future research should extend these findings using connected speech, predictive paradigms, or naturalistic listening tasks [90,91].

Finally, the relatively small sample size and multilevel analytical approach limit our ability to model individual differences comprehensively. Although we aim to collect data on ASD symptom severity (GARS-2), age, and gender, these variables could not be fully included as covariates in formal models due to power constraints. They can be considered in exploratory analyses. Notably, the sample is gender-imbalanced (28 males, 2 females), reflecting the higher male prevalence in ASD but potentially limiting generalizability, especially given known sex differences in auditory processing and ASD phenotypes. A central challenge in studying auditory and speech processing in ASD is the substantial heterogeneity within the population. Children with ASD exhibit a wide range of language outcomes, from minimally verbal to age-appropriate or even advanced language skills. Similarly, auditory hypersensitivity (e.g., distress to loud sounds) is a common sensory feature in ASD, but not universal. Some individuals may show a preference for nonspeech sounds over speech and others may not. This variability is not merely noise to be controlled for; it may be central to understanding the diverse profiles of speech perception. Future studies with larger, demographically balanced samples and integrated behavioral assessments will be essential to validate group/subgroup differences as well as individual differences and extend these findings.

## Figures and Tables

**Table 1:** Tentative Predictions for the Seven Stimulus Conditions (Based on EPF/WCC/NCH) Condition.

	Processing Type	Prediction in ASD	EPF + NCH Account	WCC + NCH Account
Consonant only	Local	↑ or = MMN amplitude ↓ or = MMN latency	Enhanced perception of phonetic detail	Intact
Vowel only	Local	↑ or = MMN amplitude ↓ or = MMN latency	Enhanced formant processing	Intact
Tone only	Global	↓ MMN amplitude ↑ MMN latency	Reduced prosodic integration	Impaired due to global integration load
C + V	Mixed/Global	↓ MMN amplitude ↑ MMN latency	Disruption due to multiple feature load	Integration weakness
V + T	Global	↓↓ MMN amplitude ↑↑ MMN latency	Disrupted holistic processing	Poor feature binding
C + T	Global	↓↓ MMN amplitude ↑↑ MMN latency	Disrupted holistic processing	Failure to form unified percept
C + V + T	Global	↓↓↓ MMN amplitude ↑↑↑ MMN latency	Collapsed integration under complexity	Strongest integration failure

Note: ↑ represents significant increase. ↓ represents significant decrease. = represents similar results when comparing MMN responses in ASD group with those in the TD group. Double/Triple arrows indicate magnitude increment/decrement.

**Table 2:** Specific Predictions Based on MMN Literature-Based Predictions for Each Stimulus Condition.

Condition	MMN Amplitude in ASD	Latency in ASD	Reduced Left-Hemispheric MMN Dominance
Consonant only	↓	↑	Predicted—based on broader speech processing literature
Vowel only	= or ↓	↑	Predicted—but may be less pronounced than for consonants
Tone only	↓	= or ↑	Predicted—particularly for lexical tones
C + V	↓↓	↑↑	Predicted—likely pronounced
V + T	↓↓	↑↑	Predicted—key for tonal languages
C + T	↓↓	↑↑	Predicted—reflects integration challenge
C + V + T	↓↓↓	↑↑↑	Predicted—most pronounced reduction expected

Note: ↑ represents significant increase. ↓ represents significant decrease. = represents similar results when comparing MMN responses in ASD group with those in the TD group. Double/Triple arrows indicate magnitude increment/decrement.

**Table 3:** Phonological Stimuli: Standard vs. Deviant Feature Variations.

Stimulus Type	Stimulus	Feature(s) Changed
Standard	/ba1/	None
Deviant 1	/ba4/	Tone change
Deviant 2	/bu1/	Vowel change
Deviant 3	/da1/	Consonant change
Deviant 4	/bu4/	Tone + Vowel Change
Deviant 5	/da4/	Tone + Consonant Change
Deviant 6	/du1/	Vowel + Consonant Change
Deviant 7	/du4/	Tone + Vowel + Consonant Change

## Data Availability

The data presented in this study are available on request from the corresponding author due to IRB requirements for data management and regulation.
